# Safety of transrectal ultrasound-guided prostate biopsy in patients receiving aspirin: An update meta-analysis including 3373 patients

**DOI:** 10.1097/MD.0000000000026985

**Published:** 2021-08-27

**Authors:** Di Chen, Gang Liu, Yurun Xie, Changsheng Chen, Zhihua Luo, Yujun Liu

**Affiliations:** a Department of Surgery, Nanxishan Hospital of Guangxi Zhuang Autonomous Region, Guilin, Guangxi, China; b The Reproductive Hospital of Guangxi Zhuang Autonomous Region, Nanning, China; c Department of Urology, People's Hospital of Guangxi Zhuang Autonomous Region, Nanning, Guangxi, China.

**Keywords:** aspirin, biopsy, cancer, meta-analysis, prostate

## Abstract

**Background:**

The management of aspirin before transrectal prostate puncture-guided biopsy continues to be controversial. The conclusions in newly published studies differ from the published guideline. Therefore, an updated meta-analysis was performed to assess the safety of continuing to take aspirin when undergoing a transrectal ultrasound-guided prostate biopsy (TRUS-PB).

**Methods:**

We searched the following databases for relevant literature from their inception to October 30, 2020: PubMed, EMBASE, Cochrane Central Register of Controlled Trials, Medline, Web of Science, Sinomed, Chinese National Knowledge Internet, and WANGFANG. Studies that compared the bleeding rates between aspirin that took aspirin and non-aspirin groups were included. The quality of all included studies was evaluated using the Newcastle-Ottawa Scale. Revman Manger version 5.2 software was employed to complete the meta-analysis to assess the risk of hematuria, hematospermia, and rectal bleeding.

**Results:**

Six articles involving 3373 patients were included in this meta-analysis. Our study revealed that compared with the non-aspirin group, those taking aspirin exhibited a higher risk of rectal bleeding after TRUS-PB (risk ratio [RR] = 1.27, 95% confidence interval [CI] [1.09–1.49], *P* = .002). Also, the meta-analysis results did not reveal any significant difference between the 2 groups for the risk of hematuria (RR = 1.02, 95%CI [0.91–1.16], *P* = .71) and hematospermia (RR = 0.93, 95%CI [0.82–1.06], *P* = .29).

**Conclusion:**

Taking aspirin does not increase the risk of hematuria and hematospermia after TRUS-PB. However, the risk of rectal bleeding, which was slight and self-limiting, did increase. We concluded that it was not necessary to stop taking aspirin before undergoing TRUS-PB.

## 1 Introduction

Prostate cancer accounts for 15% of all male tumors and is one of the most common malignant tumors of the male urinary system.^[1]^ Recently, it has been reported that the incidence and mortality of prostate cancer have declined or stabilized.^[2]^ To detect potential prostate cancer, imaging, assessment for tumor biomarkers, and prostate biopsy are commonly used in current hospital protocols. Transrectal ultrasound-guided systematic prostate biopsy is the gold standard to diagnose prostate cancer.^[3]^ However, prostate biopsy is an invasive surgical procedure that can be associated with a number of postoperative complications, including infection and bleeding.

Remarkably, prostate cancer and cardio-cerebrovascular disease often occur together in the elderly.^[4]^ Aspirin is a common anticoagulant that is widely used to prevent and treat cardio-cerebrovascular diseases in patients. Therefore, some patients might need to continue to take aspirin during prostate biopsy. Currently, the management of anticoagulants before prostate biopsy is controversial. In the clinic, aspirin is commonly stopped 7 to 10 days before prostate biopsy to reduce the incidence of bleeding complication.^[5]^ However, some comparative studies have reported different conclusion. Two prospective studies reported that using low-dose aspirin during prostate biopsy did not increase the risk of bleeding.^[6,7]^ In 2012, a meta-analysis reported that continuing to take aspirin during prostate biopsy could significantly increase the risk of hematuria,^[8]^ but discontinuing aspirin before undergoing prostate biopsy might be unnecessary.

Meta-analysis is a useful statistical tool to overcome the limitation of different sample sizes used in individual studies and has the possibility of generating more accurate conclusions. Several related articles have been published since the 2012 meta-analysis was completed. Therefore, this study carried out an updated meta-analysis of all eligible published studies to assess the safety of transrectal ultrasound-guided prostate biopsy (TRUS-PB) in patients taking aspirin.

## 2 Methods

### 2.1 Search strategy

We searched the following databases for relevant literature from their inception to October 30, 2020: PubMed, EMBASE, Medline, Cochrane Central Register of Controlled Trials, Web of Science, Sinomed, Chinese National Knowledge Internet, and WANGFANG using the PRISMA guidelines.^[9]^ All studies that reported the outcomes of interest were included. Also, the reference from all relevant publications was examined. The English search term that were used included “Biopsy” OR “Biopsies” AND “Prostate” OR “Prostates” AND “Aspirin” OR “Acetylysalicylic Acid” OR “Acylpyrin” OR “2-(Acetyloxy)benzoic Acid.”

### 2.2 Selection criteria

The following inclusion criteria were used:

(1) The published article was written in English or Chinese.
(2) The full text of the article was available.
(3) The types of studies were randomized controlled trials (RCTs) or prospective studies.
(4) All the patients included in the study underwent ultrasound-guided transrectal prostate biopsy. The patients in each study who continued to take aspirin or stopped taking aspirin during the biopsy were assigned to 1 of 2 groups, respectively.
(5) Sufficient data were provided for the meta-analysis, including the total number of subjects and outcome indicators.

Several exclusion criteria were used, including

(1) lacking the relevant and
(2) the study did not involve the use of aspirin or TRUS-PB.

### 2.3 Quality assessment of the included studies

The quality of the studies’ methods was assessed using the Newcastle-Ottawa Scale (NOS)^[10]^ and the Review Manager version 5.2 software (https://community.cochrane.org/help/tools-and-software/revman-5). Three components are assessed with NOS, including selection (0–4 points), comparability (0–1 points), and outcome (0–3 points). A NOS scores of 6 or higher were used to indicate high-quality studies. Two reviewers independently performed this analysis. Any disagreements were resolved by consensus.

### 2.4 Data extraction

Two reviewers examined the studies included in this meta-analysis. They extracted the following data: first author, publication year, study period, country, follow-up, study design, total number of patients, number of patients on follow-up, the number of needle passes, the aspirin dose, and relevant outcome indicators. The included outcomes were hematuria, rectal bleeding, and hematospermia.

### 2.5 Statistical analysis

This meta-analysis was performed using Review Manager Software (Revman Manger version 5.2, Cochrane Collaboration, Oxford, UK). Pooled risk ratios (RRs) were calculated as the summary statistic for dichotomous variables. The RRs were reported with 95% confidence intervals (CIs). The *Z* test was used to determine the pooled effects of all included studies. Statistical significance was considered to exist when the *P*-value was less than .05. Statistical heterogeneity among studies was assessed using the Cochrane Chi-squared test and inconsistency (*I*^2^). The presence of heterogeneity was considered to be significant When *P*-value less than .05 or *I*^2^ greater than 50%. A random-effects model was used to pool data when the results indicated the presence of significant heterogeneity. Otherwise, a fixed-effects model was used. Sensitivity analysis was performed to assess any significant heterogeneity and the stability of the results. Furthermore, a funnel plot was used to assess publication bias as the number of included studies was less than 10.

## 3 Results

### 3.1 Study identification

**Figure 1:**
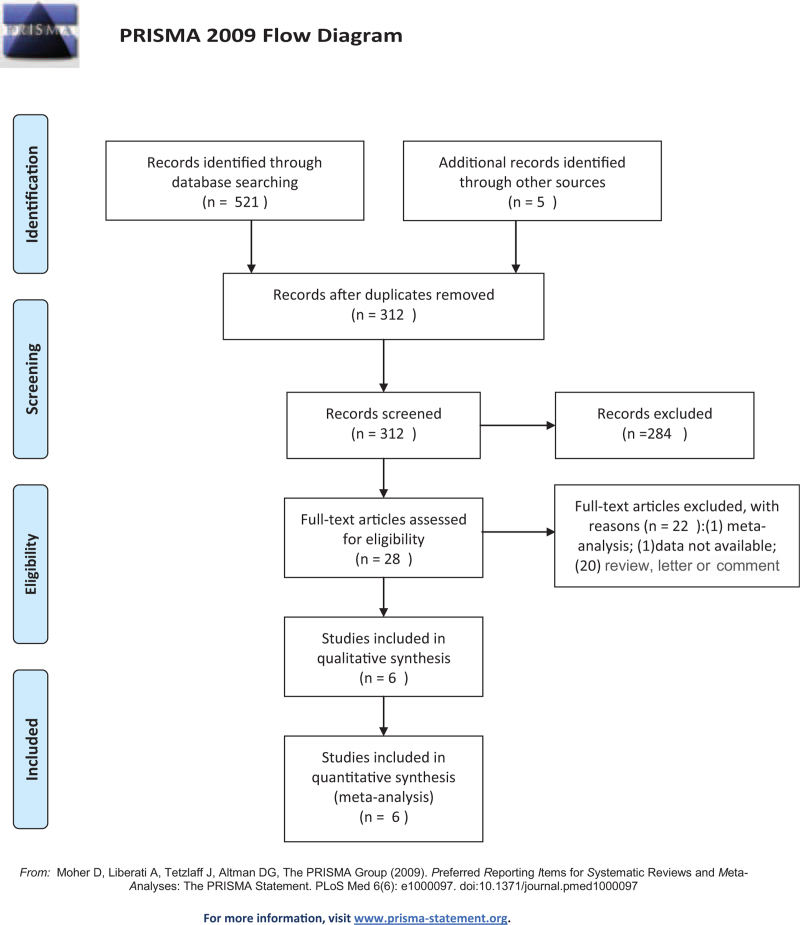
Flow diagram.

The search protocol and the results are shown in Figure 1. The initial literature search identified 526 potentially relevant studies. Two hundred fourteen studies were excluded due to duplication. Ultimately, 6 eligible studies,^[6,7,11–14]^ with a total of 1047 patients taking aspirin and 2326 control patients, were included in this meta-analysis based on the defined selection criteria.

### 3.2 Characteristics and quality

**Figure 2:**
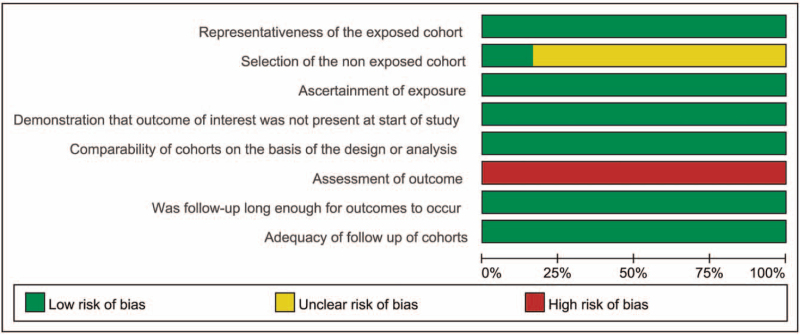
Risk of bias graph.

**Figure 3:**
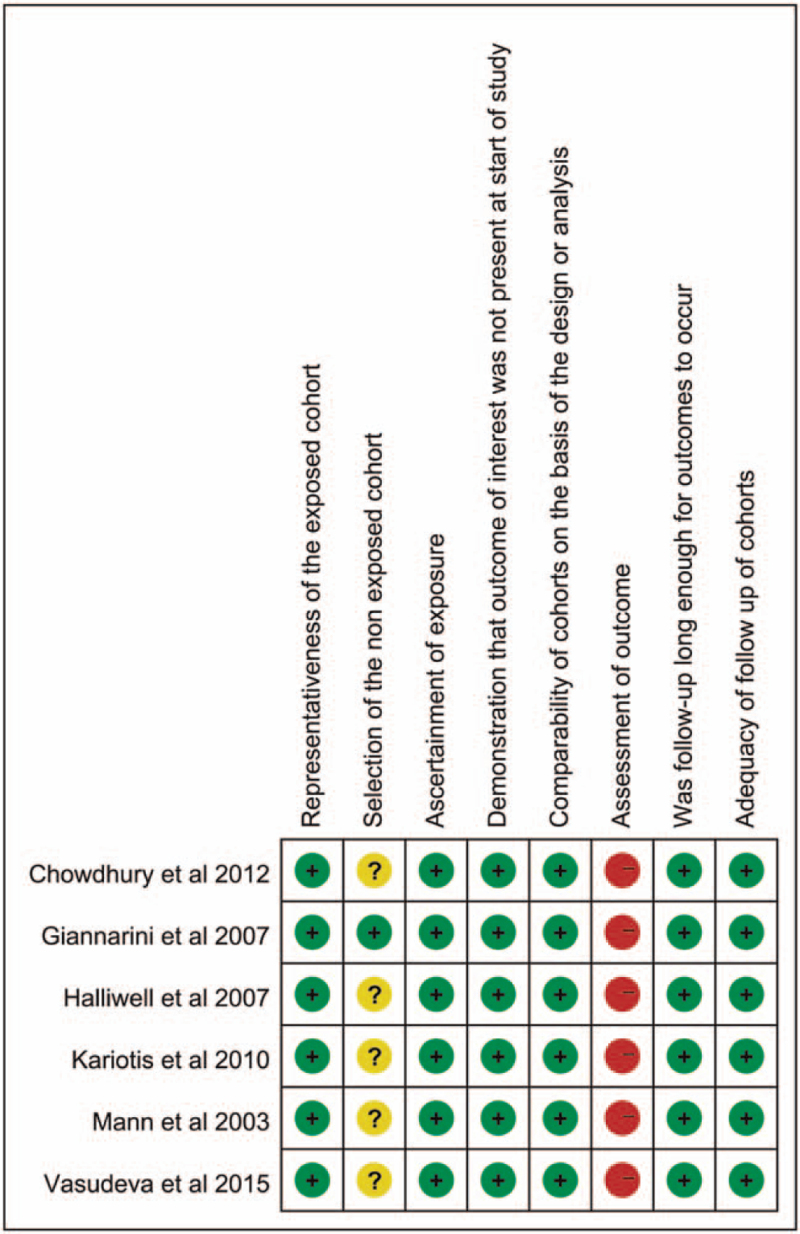
Risk of bias summary.

Table 1 summarizes the baseline characteristics of the included studies. The 6 studies included 1 RCT^[7]^ and 5 prospective cohort studies (PCS). The methodological quality of these 6 studies was high, as indicated by a NOS score of 6 to 7 out of 9 points, and 5 PCS were considered to be high quality.^[6,7,11–13]^ The results are presented in Figures 2 and 3.

**Table 1:** Characteristics of the included studies.

Study	Author	Year	Period	Country	Follow-up (d)	Study type	Total patients (n)	Patients on follow-up (n)	Needle passes(n)	Dose (mg/d)
1	Halliwell et al	2007	NR	UK	10–14	PCS	1811	1740	NR	NR
2	Chowdhury et al	2012	2009–2012	UK	10–14	PCS	930	902	8/9/10	NR
3	Kariotis et al	2010	2007–2008	Greece	30	PCS	530	434	12	100
4	Vasudeva et al	2015	2011–2014	India	21	PCS	783	681	12	75
5	Maan et al	2003	NR	UK	7	PCS	200	177	NR	75/150
6	Giannarini et al	2007	2005–2006	Italy	14	RCT	200	200	10	100

NR = not record, PCS = prospective cohort study, RCT = randomized controlled trial.

### 3.3 Meta-analysis results

#### 3.3.1 Hematuria

**Figure 4:**
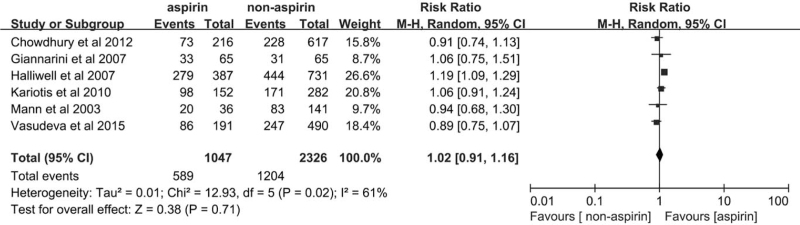
Forest plot of the hematuria of the aspirin group and the non-aspirin group.

All 6 studies reported complete data for hematuria, with 589 case of hematuria reported for patients in the aspirin group and 1204 cases of hematuria in the non-aspirin group. Our assessment of hematuria in this meta-analysis showed no significant difference between the aspirin and non-aspirin groups (RR = 1.02, 95%CI [0.91–1.16], *P* = .71, Fig. 4). However, significant heterogeneity was observed among the studies (*P* = .02, *I*^2^ = 61%).

#### 3.3.2 Rectal bleeding

**Figure 5:**
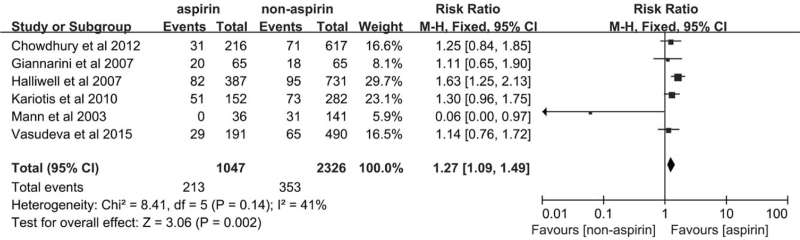
Forest plot of the rectal bleeding of the aspirin group and the non-aspirin group.

The 6 included studies reported complete data for rectal bleeding; 213 case of rectal bleeding were observed in the aspirin group, and 353 cases of rectal bleeding were reported in the non-aspirin group. Our assessment of rectal bleeding in this meta-analysis indicated that the risk for rectal bleeding in the aspirin group was significantly higher than the no aspirin group (RR = 1.27, 95%CI [1.09–1.49], *P* = .002, Fig. 5). The results for heterogeneity indicated no significant difference (*P* = .14, *I*^2^ = 41%).

#### 3.3.3 Hematospermia

**Figure 6:**
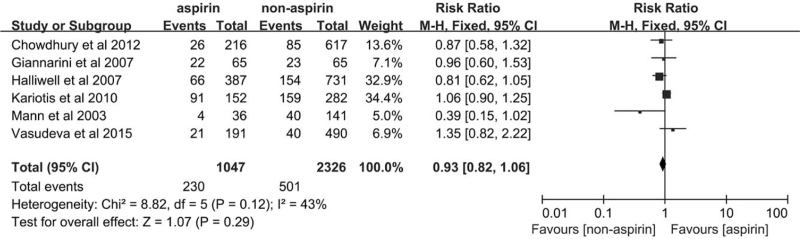
Forest plot of the hematospermia of the aspirin group and the non-aspirin group.

The included studies reported complete data for hematospermia; 203 cases of hematospermia were observed in the aspirin group, and 501 hematospermia cases were included in non-aspirin group. Our meta-analysis of hematospermia revealed no significant difference between the aspirin and non-aspirin groups (RR = 0.93, 95%CI [0.82–1.06], *P* = .29, Fig. 6). No significant heterogeneity was observed in the fixed-effect model (*P* = .12, *I*^2^ = 43%).

### 3.4 Sensitivity analysis and assessment of publication bias

To test the stability of the results and assess the possible cause of heterogeneity, we performed a sensitivity analysis using an article-by-article culling method. When the research by Halliwell et al was excluded in the analysis of hematuria, the *I*^2^ value changed from 85% to 0%, and the *P*-value changed from .02 to .57. Our results for hematuria continued to be stable, no matter which studies were removed from the analysis. Even though the pooled results for rectal bleeding did not exhibit any significant heterogeneity, the results were unstable. When the research by Halliwell et al was excluded, the result changed dramatically in that the *P*-value changed from .002 to .23. The results obtained for hematospermia were stable and did not exhibit significant heterogeneity in the analysis of sensitivity.

**Figure 7:**
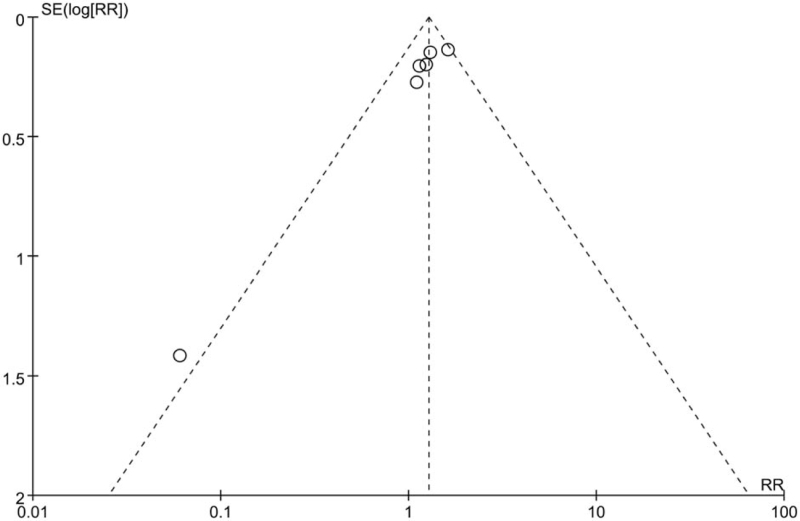
Funnel plot of the rectal bleeding.

It has been noted that Begg's test or Egger's test exhibits relatively low power to assess publication bias when included the number of included studies is less than 10. Hence, we used a funnel plot to assess publication bias. As shown in Figure 7, the study by Mann et al was located on the outer edge of the funnel plot causing the funnel plot to be unbalanced and indicated a significant publication bias for rectal bleeding in the meta-analysis.

## 4 Discussion

In the present study, our meta-analysis investigated whether continuing to take aspirin would increase the risk of bleeding after TRUS-PB. We determined that aspirin increased the risk for rectal bleeding risk, but the risk of hematuria and hematospermia did not differ from the control group. This study included 6 prospective studies, 1 RCT and 5 PCS, that enrolled 3373 patients. A follow-up report or questionnaire after the prostate biopsy was used to record each of the outcome indicators. All 6 studies reported that less than 20% of the patients were lost to follow-up. The sensitivity analysis indicated that the pooled results for hematuria and hematospermia were stable. Due to the reported statistically significant risk for rectal bleeding in most patients, the study by Hailliwell et al carried the highest weight in the pooled result for rectal bleeding.^[6]^ The results changed significantly after the study by Hailliwell et al was excluded. The remaining 5 studies reported that taking low-dose aspirin did not significantly increase the risk for rectal bleeding. Vasudeva et al performed a follow-up assessment 3 weeks after 12 core TRUS-PB.^[15]^ The result from that study indicated that compared with groups in which aspirin was not taken, taking low-dose aspirin did not increase the risk of bleeding complications. Giannarini et al carried out an RCT and reported similar questionnaire. However, they did observe that the median duration for hematuria and rectal bleeding was significantly prolonged in patients taking low-dose aspirin.^[7]^ Hailliwell et al performed a PCS that included 1811 patients over 3.5 years. They reported that taking aspirin increased the risk and duration of hematuria and rectal bleeding after TRUS-PB, but the bleeding complication that did occur was minor and self-limiting. After reviewing all 6 studies, we found that the study by Hailliwell et al included more than 700 patients and it is possible that uncertain aspirin doses resulted in different baseline characteristics that could have been associated with high bleeding risk or heterogeneity in the study.

In 2012, Carmignani et al conducted a similar meta-analysis and determined that continuing to take aspirin while undergoing a TRUS-PB might increase the risk for hematuria but did not significantly affect the risk of rectal bleeding or hematospermia.^[8]^ Our updated meta-analysis included additional related studies and performed a sensitivity analysis to assess the stability and heterogeneity of the results. Although the result obtained for rectal bleeding was unstable, no significant heterogeneity was observed in the pooled rectal bleeding results. Therefore, our results for rectal bleeding and hematospermia were persuasive because of the low heterogeneity. Thus, it might be unnecessary to discontinue aspirin use to avoid an increased risk of bleeding when undergoing a TRUS-PB.

It has been reported that aspirin primarily inhibits cyclooxygenase-1 irreversibly and reduces the synthesis of thromboxane A2, which prevents platelet aggregation and thrombosis.^[16]^ Clopidogrel, ticlopidine, and cilostazol are other antiplatelet agents used to treat or prevention of cardiovascular diseases. Anticoagulant agents, such as warfarin, dabigatran, and rivaroxaban, act as vitamin K antagonists in clinical use.^[17]^ Although many antithrombotic agents have been used, studies investigating their effects on prostate biopsy have been limited, and their conclusions were inconsistent. In 2020, Lee et al reported a consensus statement concerning prostate biopsy from the Korean Society of Urogenital Radiology.^[18]^ They reported that most doctors stopped the administration of anticoagulants or antiplatelets 1 week before a prostate biopsy due to the possible risk of higher bleeding. Similarly, the Canadian Urologic Association has recommended that antiplatelet agents or anticoagulants should be stopped before a patient undergoing a TRUS-PB. However, our meta-analysis indicated that continued administration of aspirin increased the incidence of rectal bleeding, but the risk of hematuria and hematospermia was not significantly higher than the non-aspirin group. In addition to the 6 studies included in this meta-analysis that reported similar conclusions, Raheem et al carried out a study that compared anticoagulation/antiplatelet and control groups.^[19]^ They reported that the risk of rectal bleeding in the anticoagulation/antiplatelet and control group was 40% vs 39%, respectively, but the risk for hematuria was increased in the control group. Ihezue et al reported that the severity and risk of bleeding complications were no worse in the anticoagulation group than in the control group.^[17]^

Concerning antiplatelet and anticoagulation management, transperineal ultrasound-guided prostate biopsy (TPUS-PB) appears to be similar to TRUS-PB. Saito et al performed a comparative study that included 598 patients who underwent a TPUS-PB.^[20]^ The results indicated that even though the medication group exhibited an increased bleeding risk, which was minor, it was not necessary to discontinue the use of anticoagulant or antiplatelet agents. Asano et al reported that the incidence of bleeding between group of patients who continued to use antithrombotic during TPUS-PB and a control group did not exhibit any significant differences.^[21]^ Numerous high-quality studies also have reported similar results as those seen in our meta-analysis. In 2017, study published in the EAU reported that although anticoagulant use was related to increased bleeding risks (OR: 1.28; 95%CI: 1.14–1.45; *P* < .001), the increase in overall complication risk was modest.^[3]^ Similarly, the International Consultation on Urological Disease/American Urological Association (ICUD/AUA) reported that prostate biopsy is a safe procedure for patients taking low-dose aspirin,^[22]^ which might increase the incidence of minor bleeding to approximately one-third higher than controls.

According to the Society of Interventional Radiology consensus guideline for periprocedural management of coagulation status and hemostasis risk in percutaneous image-guided interventions, the TRUS-PB was a procedure with a moderate bleeding risk including hematuria (13–58.4%), rectal bleeding (2.3–21%), and hematospermia (5–28%).^[17]^ Although these bleeding complications were reported to self-limiting, adverse events that could be associated with anticoagulant discontinuation must be considered. Biondi-Zoccai et al conducted a meta-analysis that included 50,279 patients at risk for coronary artery disease.^[23]^ Their results indicated aspirin withdrawal was associated with a three-fold higher risk for major adverse cardiac events (OR = 3.14, 95%CI [1.75–5.61]). Similarly, Ferrari et al reported 1236 patients with acute coronary heart disease that 4.1% of the patients experienced the disease due to cessation of aspirin therapy.^[24]^ Therefore, aspirin withdrew is not always safer or necessary.

Our research exhibited several limitations: first, a follow-up assessment or questionnaire was used to collect the outcome data in 6 studies included in this meta-analysis. Patients might forget some details, which could lead to bias. However, it is difficult to avoid this type of bias due to this study design. Second, only 1 included study was a RCT. The addition of more high-quality RCTs would strengthen the conclusions. Third, the heterogeneity was high in the hematuria analysis. Although we observed the study generated heterogeneity, the underlying cause for heterogeneity was not clear. Finally, even though the pooled results for rectal bleeding did no exhibit significant heterogeneity, the sensitivity analysis indicated an unstable result. The inclusion of additional studies might increase the stability.

## 5 Conclusion

Our research indicated that taking aspirin did not increase the risk of hematuria and hematospermia after TRUS-PB. The risk of postoperative rectal bleeding did increase, but the bleeding was minimal and self-limiting. Therefore, we concluded that it is not necessary to stop taking aspirin before TRUS-PB. However, additional high-quality randomized controlled studies are needed to support this conclusion.

## Acknowledgments

The authors would like to express their gratitude to EditSprings (https://www.editsprings.com/) for the expert linguistic services provided.

## Author contributions

Di Chen: conceived the study, participated in its design, coordinated, and drafted the manuscript.

Gang Liu: article review, data extract, and manuscript revising; Yurun Xie: data extract and performed the bias evaluation. Changsheng Chen: data extract and performed the bias evaluation. Zhihua Luo: manuscript revising. Yujun Liu: design, project development, supervisior, and manuscript revising. All authors read and approved the final manuscript.

**Conceptualization:** Yujun Liu.

**Data curation:** Yurun Xie, Zhihua Luo.

**Investigation:** Changsheng Chen.

**Methodology:** Di Chen.

**Software:** Changsheng Chen.

**Supervision:** Gang Liu.

**Writing – original draft:** Di Chen.

**Writing – review & editing:** Gang Liu.
